# Research on the Hippo Pathway in Cancer

**DOI:** 10.3390/cells15090833

**Published:** 2026-05-01

**Authors:** Fengqiu Dang, Shuhuan Dai, Tianqi Zhao, Rong Zhang, Long Chen, Yongxiang Zhao

**Affiliations:** 1 Pharmaceutical College, Guangxi Medical University, Nanning 530021, China; dangfengqiu@sr.gxmu.edu.cn; 2 State Key Laboratory of Targeting Oncology, National Center for International Research of Bio-Targeting Theranostics, Guangxi Key Laboratory of Bio-Targeting Theranostics, Collaborative Innovation Center for Targeting Tumor Diagnosis and Therapy, Guangxi Talent Highland of Major New Drugs Innovation and Development, Targeting Theranostics Research Center of Guangxi Higher Education, Guangxi Medical University, Nanning 530021, China; 202421687@sr.gxmu.edu.cn (S.D.); 202421684@sr.gxmu.edu.cn (T.Z.); zhangrong@sr.gxmu.edu.cn (R.Z.); longchen@sr.gxmu.edu.cn (L.C.)

**Keywords:** Hippo, YAP/TAZ, tumor microenvironment, cancer-associated fibroblasts

## Abstract

**Highlights:**

**What are the main findings?**

- Hippo/YAP/TAZ activation drives tumor proliferation, stemness, metabolism, and drug resistance.
- Hippo regulates CAF activation and immunosuppression, while TME cues reciprocally promote Hippo activity, forming a pro-tumorigenic loop.

**What are the implications of the main findings?**

- Hippo–TME crosstalk provides a mechanistic basis for combinatorial anti-cancer strategies.
- TEAD palmitoylation inhibitors and YAP–TEAD disruptors in clinical trials offer precision options for Hippo-dysregulated tumors.

**Abstract:**

The Hippo, as a central pathway regulating cell proliferation, apoptosis, stem cell homeostasis and organ development, is closely associated with the onset and progression of tumors, metabolic reprogramming, drug resistance and immune evasion when it is abnormally inactivated. The Hippo not only directly promotes tumor cell proliferation, maintains cancer stem cell properties, and mediates metabolic reprogramming and treatment resistance, but also reshapes the tumor microenvironment (TME) by regulating the formation, heterogeneity and function of cancer-associated fibroblasts (CAFs). Furthermore, it mediates tumor immunosuppression and immune evasion by modulating programmed death-ligand 1 (PD-L1) expression, T-cell function, macrophage polarization and cytokine secretion. At the same time, inflammatory cytokines, growth factors, metabolites and physical signals within the TME can negatively regulate the activity of the Hippo, creating a pro-tumor positive feedback loop. This article provides a systematic review of the composition and regulation of the Hippo, its mechanisms of action in the biological behavior of tumor cells and interactions within the tumor microenvironment, as well as progress in the development of drugs targeting this pathway. It offers a theoretical basis for a deeper understanding of the role of the Hippo in tumors and for the development of novel anti-tumor therapeutic strategies.

## 1. The Composition and Regulation of the Hippo Pathway 

### 1.1. Classic Regulation of the Hippo Pathway 

Cancer, as one of the major diseases threatening human health, is subject to complex regulatory influences from a variety of factors in its onset and progression. In particular, the Hippo pathway has become a major focus in cancer research due to its crucial role in regulating key biological processes such as organ size, cell proliferation, apoptosis and stem cell self-renewal [1]. The Hippo pathway was first discovered in fruit flies; its core components include the upstream kinases mammalian STE20-like kinase 1/2 (MST1/2), the scaffold protein Salvador homolog 1 (SAV1), co-activators Mps One Binder kinase activator-like 1A/1B (MOB1A/B), downstream kinases large tumor suppressor 1/2 (LATS1/2) and downstream effector molecules Yes-associated protein/transcriptional co-activator with PDZ-binding motif (YAP/TAZ) [2]. This pathway regulates the phosphorylation status, nuclear translocation and transcriptional activity of YAP/TAZ, thereby influencing the expression of downstream genes (such as CTGF, CYR61 and ANKRD1) and ultimately regulating cell proliferation, apoptosis and the maintenance of stemness [3]. When the Hippo pathway is activated, MST1/2 activates LATS1/2 through phosphorylation; LATS1/2 subsequently phosphorylates YAP/TAZ, causing them to bind to 14-3-3 proteins, remain in the cytoplasm, and be inactivated via proteasomal degradation. When the Hippo pathway is inactivated, unphosphorylated YAP/TAZ translocates to the cell nucleus, where it binds to transcription factors of the transcriptional Enhancer factor domain family members 1-4 (TEAD1-4), driving the transcription of downstream target genes, thereby promoting cell proliferation, inhibiting apoptosis and maintaining stemness. However, the role of the Hippo pathway in tumor initiation and progression is far from straightforward; a growing body of research suggests that it is also closely linked to the tumor microenvironment and tumor metabolism [4].

### 1.2. Signaling Network of the Hippo Pathway

**Figure 1:**
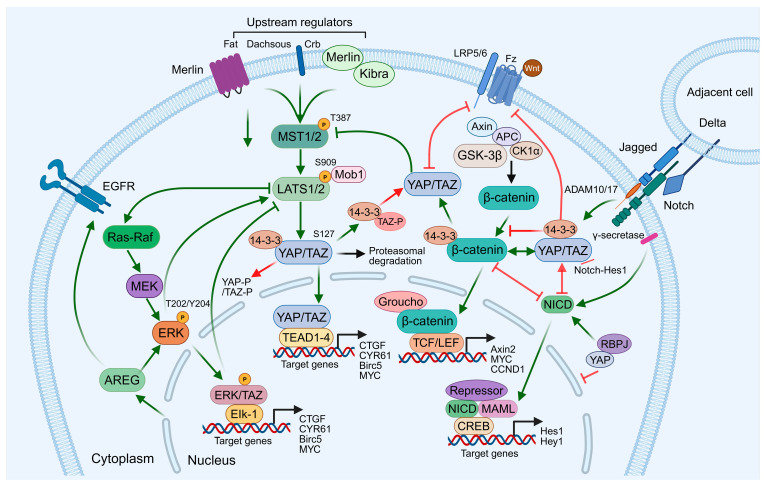
Schematic diagram of the Hippo pathway and its crosstalk with EGFR-MAPK, Wnt/β-catenin, and Notch signaling pathways.

The classical regulation of the Hippo pathway is also modulated by protein ubiquitination, phosphorylation and various upstream signals, forming an interconnected network through multiple signals and environmental factors (Figure 1). These include cell polarity, mechanical stress, cell density, soluble factors and stress signals. For example, the RING-type E3 ubiquitin ligase RNF214 can non-degradatively ubiquitinate TEADs, enhancing the TEAD-YAP/TAZ interaction and thereby promoting the expression of Hippo pathway downstream genes [5,6]. At the level of growth factors, EGFR signaling can bypass RhoA via the PI3K-PDK1 pathway to directly activate YAP, thereby enhancing the expression of its downstream target genes (such as CYR61) and promoting the proliferation of hepatocellular carcinoma; the combined inhibition of EGFR and YAP produces a synergistic cytotoxic effect [7]. At the level of development and stem cell signaling, YAP interacts directly with Wnt/β-catenin to form a transcriptional complex within the nuclei of intestinal epithelial cells, regulating Wnt target genes such as Lgr5 and cyclin D1, thereby promoting cellular self-renewal, regeneration and the development of inflammation-associated tumors. Furthermore, in the liver, Hippo pathway deficiency activates a positive feedback loop between YAP/TAZ and Notch, which enhances Notch signaling via JAG1, thereby promoting excessive liver growth and the rapid development of HCC; conversely, activation of the Wnt/β-catenin pathway partially inhibits this positive feedback loop, highlighting the complex regulatory interactions between Hippo pathway, Notch and Wnt [8]. Beyond chemical cues, the Hippo pathway integrates physical and mechanical signals. YAP/TAZ senses matrix stiffness, fluid shear stress, cell morphology, and interstitial fluid pressure, thereby modulating TEAD-dependent transcription and cell volume regulation, and establishing feedback loops to maintain cellular homeostasis [9]. This mechanism highlights that the Hippo pathway functions not only as a central signal integrator but also as a sensor and regulator of microenvironmental dynamics.

Collectively, through its interactions with EGFR, Wnt/β-catenin, Notch, and mechanical or microenvironmental cues, the Hippo pathway orchestrates a sophisticated signaling network. This network is pivotal for tissue development, stem cell homeostasis, regeneration, and tumorigenesis, serving as a central hub for cell fate determination and multi-signal integration. It underscores the multilayered complexity inherent to canonical Hippo pathway regulation.

## 2. Hippo Pathway and Tumor Cell Biological Behaviors

### 2.1. Promotion of Tumor Cell Proliferation and Cancer Stem Cell Traits

Aberrant activation of the Hippo pathway downstream effectors YAP and TAZ is recognized as a central driver of tumor proliferation and the maintenance of cancer stem cell (CSC) traits [10]. CSCs constitute a subpopulation endowed with self-renewal, multilineage differentiation, and potent tumor-initiating capacity. YAP/TAZ directly regulate stemness-associated genes, including *Oct4*, *Sox2*, and *Nanog*, which are critical for sustaining CSC self-renewal and plasticity. By activating these programs, YAP/TAZ can reprogram non-stem tumor cells into CSCs, expanding the CSC pool and thereby promoting tumor initiation and progression [2].

Cell-cell adhesion molecules, such as E-cadherin and their associated α/β-catenin, modulate the Hippo pathway via mechanical tension and adhesion complexes, enforcing contact inhibition [10]. Disruption of adhesion or alterations in mechanical stress can activate YAP/TAZ, sustaining tumor proliferation and CSC traits [11,12]. In pancreatic ductal adenocarcinoma, macrophage-derived CCL5 enhances tumor stemness through the CCR5/AKT/Sp1/CD44 axis, while tumor cell-secreted AREG stimulates macrophage CCL5 production via the Hippo pathway-YAP, establishing a positive feedback loop [13]. In oral squamous cell carcinoma, CAF-derived lactate stabilizes YAP1 phosphorylation by inhibiting MST1 ubiquitination and degradation, further reinforcing CSC maintenance [14].

In squamous cell carcinoma, partial epithelial–mesenchymal transition (pEMT) is orchestrated by the Hippo pathway, Notch, and transforming growth factor-β (TGF-β) signaling, as well as by microRNAs, lncRNAs, and the tumor microenvironment, collectively enhancing invasiveness and stem-like traits [15]. Hippo pathway dysregulation induced by *Helicobacter pylori* infection similarly drives gastric cancer cell proliferation. Highly stem-like malignant epithelial cells exhibit more complex intercellular communication and dedifferentiation features, with the Hippo pathway serving as a key determinant of functional differences between high- and low-stemness populations [16].

### 2.2. Involvement in Tumor Cell Metabolic Reprogramming

The Hippo pathway downstream effectors YAP/TAZ play a pivotal role in tumor metabolic reprogramming, coordinating energy production, amino acid metabolism, and biosynthesis to sustain rapid proliferation. YAP/TAZ upregulate the glutamine transporter ASCT2/SLC1A5 and glutaminases GLS1/2, enhancing glutamine uptake and hydrolysis, and converting glutamate to α-ketoglutarate for entry into the tricarboxylic acid cycle, thereby supplying ATP and biosynthetic precursors for lipid, nucleotide, and protein synthesis [17]. YAP activation further promotes glutamine metabolism by stimulating CTGF secretion and extracellular matrix deposition, while suppressing autophagy via the mTORC1 axis to maintain energy homeostasis and a growth advantage [18,19]. In prostate cancer, MYBL2 inhibits the Hippo pathway upstream kinase LATS1, enhancing YAP nuclear localization and transcriptional activity, thereby driving metabolic gene expression and supporting proliferation and bone metastasis under androgen-deprived conditions [20]. Moreover, YAP/TAZ regulate fatty acid metabolism, glutamine-derived α-KG production, and ROS homeostasis, conferring a survival advantage under nutrient scarcity and oxidative stress [21]. Collectively, these findings reveal a tight coupling between Hippo pathway/YAP signaling and glutamine- and lipid-driven metabolic networks in cancer cells.

### 2.3. Mediation of Tumor Cell Drug Resistance

Tumor drug resistance remains a major challenge in cancer therapy, and dysregulation of the Hippo pathway—a central regulator of cell fate—is closely linked to resistance across multiple malignancies [22]. In pancreatic cancer, Hippo pathway/YAP signaling mediates gemcitabine resistance through complex crosstalk between tumor cells and tumor-associated macrophages. Macrophage-derived CCL5 activates the CCR5/AKT/Sp1/CD44 axis in cancer cells, enhancing stemness and chemoresistance. Reciprocally, tumor cell-secreted AREG stimulates macrophage CCL5 production via the Hippo pathway-YAP, establishing a positive feedback loop that sustains drug-resistant states. Disruption of this loop, for instance by Sp1 inhibition with Mithramycin, sensitizes tumors to gemcitabine [23].

Moreover, SP1-driven miR-31-5p suppresses LATS2 and inhibits the Hippo pathway in pancreatic cancer cells (PCCs), while extracellular vesicle–mediated transfer of miR-31-5p to pancreatic stellate cells activates SPARC secretion, which in turn stimulates ERK signaling in cancer cells, promoting chemoresistance. In intrahepatic cholangiocarcinoma, SAV1 loss-of-function mutations repress the Hippo pathway, leading to sustained YAP activation, increased proliferation, and resistance to targeted therapies [24].

YAP/TAZ further reinforce resistance by regulating drug efflux pumps, apoptosis inhibitors, and metabolic adaptation pathways, while synergizing with PI3K/AKT, MAPK, and NF-κB signaling to enhance DNA repair and oxidative stress tolerance, stabilizing the resistant phenotype [25]. Downstream effectors of the Hippo pathway, YAP and TAZ, play a pivotal role in tumor immune resistance. Aberrant activation of the YAP/TAZ–TEAD axis promotes extracellular matrix remodeling, suppresses CD8^+^ T cell infiltration, and enhances the secretion of immunosuppressive factors, thereby directly driving immune evasion and diminishing the efficacy of anti-PD-1/PD-L1 therapies [26]. In BRAFi-resistant melanoma, intrinsic YAP is markedly activated, and its interaction with TEAD directly upregulates PD-L1 transcription, impairing CD8^+^ T cell cytotoxicity and cytokine production. Clinical specimens further reveal that tumors with high nuclear YAP localization exhibit elevated PD-L1 expression, highlighting the YAP–PD-L1 signaling axis as a central mechanism underlying BRAFi resistance-associated immune escape and suggesting that combinatorial strategies targeting YAP or immune checkpoints may enhance therapeutic outcomes [27].

RET is a receptor tyrosine kinase whose fusions or mutations drive tumorigenesis in non-small cell lung cancer and thyroid cancer. Studies have shown that RET-TKI treatment activates the Hippo pathway downstream effector YAP in tumor cells; nuclear YAP binds TEAD to transcriptionally upregulate HER3, restoring ERK signaling and sustaining cell proliferation, thereby driving adaptive resistance to RET-TKIs [28]. In cancer models harboring KRAS G12C or G12D mutations, both acquired and intrinsic resistant cells exhibit enhanced nuclear localization and activation of YAP/TAZ. Upon exposure to KRAS inhibitors, YAP/TAZ are rapidly activated, preventing drug-induced apoptosis and engaging the SLC7A5/mTORC1 axis to maintain proliferation, leading to resistance [25]. In BRAF-mutant tumors—including melanoma, colorectal cancer, and non–small cell lung cancer—YAP activation functions as a parallel survival input to RAF–MEK signaling, diminishing the efficacy of RAF or MEK inhibitors such as vemurafenib and trametinib [29].

On one hand, the Hippo pathway enhances tumor cell proliferation, suppresses apoptosis, and activates compensatory survival pathways, thereby reducing the efficacy of chemotherapy and targeted therapies. On the other hand, by modulating the tumor microenvironment through positive feedback loops and immunosuppressive signals, it limits CD8^+^ T cell infiltration and sustains resistance. Combined interventions targeting the Hippo pathway/YAP/TAZ–TEAD axis or the associated microenvironmental cues may improve the efficacy of chemotherapy, targeted therapy, and immunotherapy, providing a mechanistic rationale for precision oncology strategies.

### 2.4. Mediation of Tumor Cell Proliferation, Apoptosis, and Transformation

YAP/TAZ regulate the expression of cell cycle-associated genes, including *CCND1*, *CDK1*, and *c-MYC*, accelerating cell cycle progression and driving tumor cell proliferation [30]. Across multiple cancers, YAP/TAZ overexpression positively correlates with proliferation rates [31]. Concurrently, Hippo pathway dysregulation confers resistance to apoptosis: YAP/TAZ activation upregulates anti-apoptotic genes, such as BCL2 family members, suppresses apoptotic markers, including cleaved Caspase-3 and cleaved PARP, and modulates cellular metabolism and ROS homeostasis to reduce sensitivity to apoptotic signals [32].

In hepatocellular carcinoma (HCC), elevated SNORD88B anchors WRN in the nucleolus to repress *MST1* (STK4) transcription, inactivating the Hippo pathway, promoting nuclear YAP accumulation, and activating pro-proliferative transcriptional programs, thereby enhancing tumor cell proliferation and malignant potential. SNORD88B loss markedly inhibits tumor formation [33]. In prostate cancer, MYBL2 upregulation suppresses LATS1 activity and activates RhoA, enhancing YAP nuclear localization, triggering YAP/TAZ transcriptional programs, and promoting proliferation while inhibiting apoptosis. MYBL2 deletion or pharmacological YAP/TAZ inhibition further induces apoptotic responses [20].

Collectively, Hippo pathway/YAP signaling integrates transcriptional control, cell cycle regulation, apoptosis suppression, and metabolic adaptation to drive tumor proliferation, survival, and malignant transformation. Therapeutic interventions targeting YAP/TAZ or upstream regulators—including LATS1 activators, RhoA inhibitors, or MYBL2-directed strategies—may offer effective approaches to restrain tumor growth and reverse malignant phenotypes.

## 3. Crosstalk Between the Hippo Pathway and the Tumor Microenvironment

TME is a dynamic and complex ecosystem comprising tumor cells, immune cells, endothelial cells, stromal cells, and the extracellular matrix, whose intricacy directly shapes tumor proliferation, invasion, metastasis, and therapeutic response [34]. Within the TME, CAFs represent the most abundant stromal cell population. Beyond their roles in extracellular matrix (ECM) production, remodeling, and tissue stiffness regulation, CAFs engage in extensive signaling crosstalk with tumor and immune cells through the secretion of cytokines, chemokines, and growth factors, thereby shaping either pro- or anti-tumor microenvironments. CAFs participate in complex feedback networks with cancer and immune cells: they suppress CD8^+^ T cell activity via TGF-β secretion and simultaneously promote tumor proliferation, invasion, and chemoresistance [35]. Meanwhile, immune cells within the TME, including macrophages, dendritic cells, myeloid-derived suppressor cells, and T cells, collectively influence the dynamic balance between tumor immune surveillance and immune evasion by regulating inflammation, anti-tumor immunity, and immunosuppression. Endothelial cells further modulate the local microenvironment and therapeutic response through their roles in tumor angiogenesis, vascular permeability, and immune cell recruitment. Increasing evidence indicates that the Hippo pathway plays a pivotal role in shaping CAF heterogeneity and remodeling the TME [36]. In particular, Hippo pathway/YAP signaling connects intrinsic tumor cell programs with immune regulation in the TME by controlling CAF subpopulation emergence and secretory profiles, thereby coordinating tumor growth, immune escape, and therapeutic resistance. Accordingly, the following sections focus on the regulatory roles of the Hippo pathway in CAF formation, activation, and heterogeneity.

CAFs exhibit functional and phenotypic heterogeneity: myofibroblastic CAFs (myCAFs) express high levels of α-SMA and are involved in mechanical signaling and ECM remodeling [37]; inflammatory CAFs (iCAFs) primarily secrete pro-inflammatory factors [38]; antigen-presenting CAFs (apCAFs) modulate T cell differentiation and immune responses [20,39]. Increasing evidence highlights a pivotal role for the Hippo pathway in CAF heterogeneity and TME remodeling. In mouse breast cancer models, activation of LATS1/2 in tumor cells induces NCAM1^+^αSMA^+^ myCAF-like subsets that overexpress TGFβ1/2, Thbs1, and Mmp14, suppress CD8^+^ T cell activity, and promote immune evasion and tumor growth, whereas LATS1/2 loss reduces this CAF population, enhances CD8^+^ T cell function, and diminishes immunosuppression. The Hippo pathway also drives the conversion of normal fibroblasts into immunosuppressive CAFs through tumor cell-secreted factors such as TGFβ and CTGF, reinforcing the immunosuppressive TME [40].

Within the TME, CAFs form complex feedback networks with tumor and immune cells: they inhibit CD8^+^ T cell activity via TGFβ secretion and concurrently promote tumor proliferation, invasion, and chemoresistance [41]. By regulating CAF subset composition and secretome, Hippo pathway/YAP signaling links tumor-intrinsic pathways with TME immune modulation, coordinating tumor growth and immune evasion.

### 3.1. Hippo Pathway Regulation of CAF Formation and Activation

The Hippo pathway also drives the conversion of normal fibroblasts into immunosuppressive CAFs by regulating tumor cell secretion of TGF-β and CTGF, thereby reinforcing the immunosuppressive TME [40]. In tumor cells, Bcl-2 modulates LATS1/2, enhancing YAP/TAZ nuclear translocation and activating downstream transcriptional programs, which promote CAF migration and activation as well as tumor proliferation and invasion [42]. Similarly, in prostate cancer, MYBL2 upregulation inhibits LATS1 activity and activates RhoA GTPase, increasing YAP/TAZ nuclear localization, driving the secretion of pro-fibrotic factors, and inducing fibroblast conversion into activated CAFs, thereby enhancing tumor growth, invasion, and therapy resistance; inhibition of MYBL2 or YAP/TAZ reverses this process [20]. YAP/TAZ activation further promotes CAF secretion of collagen, fibronectin, and MMPs, simultaneously enhancing ECM synthesis and remodeling to regulate tumor cell migration and invasion [43]. Hippo pathway/YAP signaling interacts with TGF-β, Wnt, and other pathways to coordinately regulate CAF formation, activation, and function, linking intrinsic tumor cell programs with TME immune regulation and orchestrating tumor growth and immune evasion [44]. Distinct CAF subtypes form complex feedback networks with tumor and immune cells, suppressing CD8^+^ T cell activity while promoting tumor proliferation, invasion, and therapy resistance [41].

### 3.2. Hippo Pathway and CAF Heterogeneity

CAFs constitute a highly heterogeneous stromal population within the TME [45]. Based on phenotype and function, CAFs can be classified into distinct subtypes. MyCAFs characterized by high α-SMA and TGF-β expression, primarily mediate ECM remodeling, fibrosis, and mechanotransduction. ICAFs, with low α-SMA and high IL-6, mainly regulate immune-inflammatory responses by secreting pro-inflammatory factors [38,39]. ApCAFs modulate T cell differentiation and immune responses [20,39], while regulatory CAFs (rCAFs) contribute to immunosuppressive functions. The relative abundance and functional properties of these subtypes vary across tumor types and progression stages, reflecting the complexity of the TME [46].

**Figure 2:**
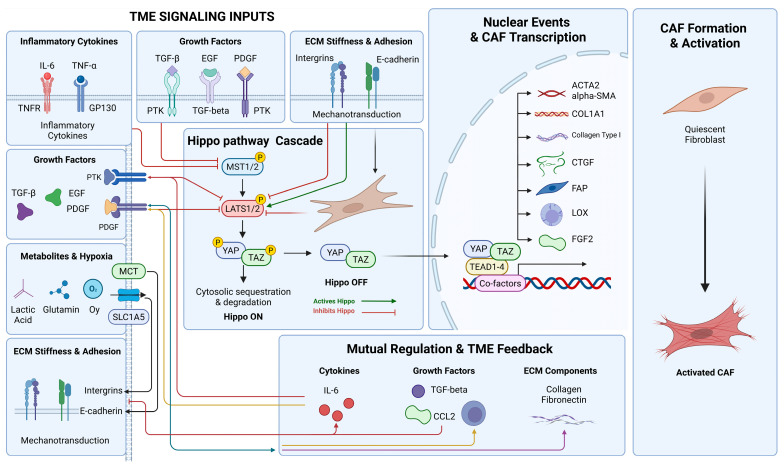
Schematic diagram of Hippo pathway regulation of CAF activation in the TME.

Upstream kinases LATS1/2 in tumor cells directly influence CAF composition. In the 4T1 murine breast cancer model, LATS1/2 activation promotes the formation of NCAM1^+^αSMA^+^ myCAFs, which express high levels of TGF-β and suppress T cell activity, enhancing immune suppression, whereas LATS1/2 loss reduces this subtype and shifts the TME toward an immunoactive state [40]. Dephosphorylated YAP/TAZ translocates to the nucleus, where it cooperates with TEAD to activate ECM remodeling and pro-fibrotic genes such as CTGF, CYR61, and LOX, while integrating RhoA GTPase signaling to couple mechanosensing with transcriptional programs [9]. Single-cell RNA sequencing further reveals that the proportions and functional characteristics of CAF subtypes—including myCAF, iCAF, apCAF, and rCAF—dynamically change under Hippo pathway regulation, indicating that the Hippo pathway integrates mechanical cues and intercellular communication to drive CAF heterogeneity through dual mechanisms [47]. In breast cancer models, LATS1/2 activation induces NCAM1^+^αSMA^+^ CAFs expressing TGF-β1/2, Thbs1, and Mmp14, exhibiting myCAF features and suppressing CD8^+^ T cell function to enhance immune evasion and tumor growth, whereas LATS1/2 deficiency diminishes this subtype, restores CD8^+^ T cell activity, and reduces immunosuppression [40] (Figure 2).

## 4. Impact of the Hippo Pathway on Immune Cell Function

Immune cell function within the TME is regulated by multiple factors, with the Hippo pathway emerging as a critical modulator of immune activity and function. This section focuses on how the Hippo pathway shapes the activity of two key immune populations—dendritic cells (DCs) and T cells—and examines its broader role in orchestrating tumor immune regulation.

### 4.1. Hippo Pathway and T Cell Suppression

The Hippo pathway, through its core effectors YAP and TAZ, plays a central role in tumor immune suppression, particularly in modulating tumor–T cell interactions via multiple mechanisms [48]. T cells, the primary effectors of adaptive immunity, comprise CD8^+^ cytotoxic T lymphocytes (CTLs), which directly kill tumor or infected cells, and CD4^+^ helper T cells, which coordinate immune responses through cytokine secretion [44].

YAP/TAZ directly regulate immune checkpoint expression, including PD-L1. In tumor cells, inhibition of upstream kinases MST1/2 or LATS1/2 leads to YAP/TAZ dephosphorylation and nuclear translocation, where they complex with TEAD transcription factors to activate the PD-L1 promoter. This upregulates PD-L1, suppresses CD8^+^ T cell proliferation and cytotoxicity, and reduces CD4^+^ T cell secretion of pro-inflammatory cytokines such as IL-2, facilitating immune evasion. In non-small cell lung cancer, CAF-derived TGF-β1 modulates YAP/TAZ activity and PD-L1 expression, influencing immunosuppressive states [49]. Conversely, RC48-ADC in bladder cancer activates Hippo pathway-TAZ signaling to downregulate PD-L1 transcription, induce secretion of CCL5, CXCL9, and CXCL14, recruit cytotoxic T cells, and restore immune surveillance, overcoming immunotherapy resistance [50]. In nasopharyngeal carcinoma bone metastases, SPHK1-generated S1P activates the Hippo pathway, expanding exhausted CD8^+^ T cells and suppressing immune defense; combined SPHK1 inhibition and anti-PD-1 therapy enhances therapeutic efficacy [51].

YAP also critically regulates regulatory T cell differentiation and function. Tregs, a CD4^+^ T cell subset expressing FOXP3, suppress excessive immune responses and maintain tolerance. In breast cancer models, YAP-high Tregs amplify FOXP3 and activin signaling, reinforcing TGF-β/SMAD-mediated immunosuppression, whereas YAP-deficient Tregs exhibit impaired function and fail to suppress antitumor immunity [52].

Moreover, YAP/TAZ activation induces tumor cell secretion of immunosuppressive cytokines and chemokines, promoting myeloid-derived suppressor cell (MDSC) and immunosuppressive macrophage infiltration, further dampening CD8^+^ T cell cytotoxicity [53]. Collectively, these multilayered mechanisms establish an immunosuppressive TME that facilitates tumor immune evasion. Targeting the Hippo pathway/YAP/TAZ axis can downregulate PD-L1, impair Treg function, and remodel suppressive immune cell infiltration, offering a promising strategy to synergize with immune checkpoint blockade therapies.

### 4.2. Role of the Hippo Pathway in PD-L1 Regulation

PD-L1 is a key immune checkpoint that binds PD-1 on T cells, suppressing their activation and effector functions, and thereby mediating tumor immune evasion [54]. The Hippo pathway regulates PD-L1 expression, directly influencing antitumor immune responses.

PD-L1 expression on tumor cells is a central mechanism inhibiting T cell activity [55], while chemokines coordinate immune cell recruitment into the tumor microenvironment [56]. RC48-ADC, a novel antibody–drug conjugate, activates Hippo pathway to suppress PD-L1 transcription, reversing HER2-driven immunosuppressive TMEs and enhancing immunotherapy efficacy. It also promotes the secretion of chemokines such as CCL5, CXCL9, and CXCL14, recruiting cytotoxic T lymphocytes and generating synergistic effects with CTLA-4 and PD-L1 blockade in preclinical models [24]. In bladder cancer, RC48-ADC modulates PD-L1 expression via Hippo pathway/YAP activation, directly impacting immunotherapy outcomes.

**Figure 3:**
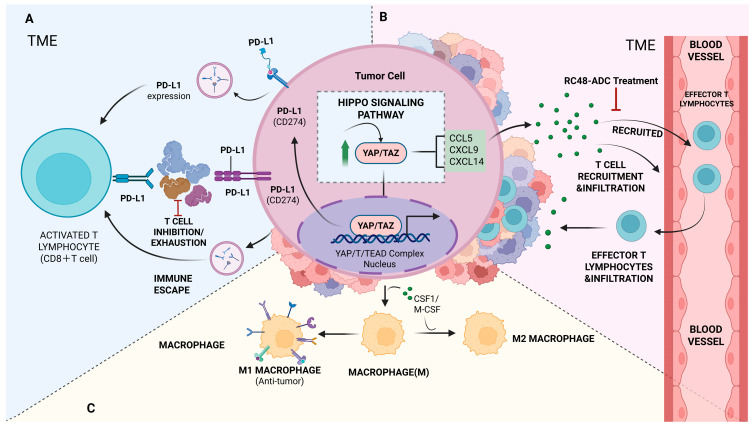
Hippo pathway in tumor immune regulation. (**A**) T cell suppression (PD-L1 upregulation and Treg amplification). (**B**) Chemokine release and macrophage polarization (CCL5/CXCL9/CXCL14 secretion and M2 polarization). (**C**) Therapeutic targeting by RC48-ADC (PD-L1 downregulation and immune restoration).

By downregulating PD-L1, Hippo pathway activation relieves tumor-mediated T cell suppression, restoring T cell activation and proliferation, and thereby potentiating antitumor immunity [37] (Figure 3A).

### 4.3. Hippo Pathway Promotes Chemokine Release

RC48-ADC treatment promotes the release of chemokines such as CCL5, CXCL9, and CXCL14, thereby facilitating the infiltration of cytotoxic T lymphocytes into the tumor microenvironment. These chemokines play pivotal roles in T cell migration and localization. Specifically, CCL5 primarily recruits T cells, natural killer cells, and dendritic cells via CCR5 engagement, whereas CXCL9 and CXCL14 attract activated T cells predominantly through CXCR3 signaling. By upregulating these chemokines, the Hippo pathway effectively directs cytotoxic T cells to tumor sites, enhancing local immune responses and ultimately promoting tumor cell elimination [57]. In bladder and nasopharyngeal carcinoma models, Hippo pathway-mediated inhibition of YAP/TAZ decreases PD-L1 transcription, relieving tumor-mediated immunosuppression, while concomitantly upregulating chemokines including CCL5, CXCL9, and CXCL14 [58]. These chemokines, through interactions with their corresponding receptors on T cells, facilitate the recruitment of CD8^+^ cytotoxic T lymphocytes and other effector immune cells, thereby amplifying local antitumor immunity. Notably, their promoter regions harbor NF-κB and IRF binding sites; Hippo pathway modulation of YAP/TAZ activity indirectly influences transcription factor occupancy at these promoters, driving chemokine expression and enhancing the recruitment of CD8^+^ T cells and NK cells (Figure 3B).

### 4.4. Impact of the Hippo Pathway on Macrophage Polarization

Macrophages constitute a critical component of the tumor microenvironment and can be broadly categorized into pro-inflammatory M1 and immunosuppressive M2 phenotypes [59]. M1 macrophages predominantly mediate antitumor immunity, whereas M2 macrophages promote tumor growth, angiogenesis, and immune suppression. The Hippo pathway plays a pivotal role in regulating macrophage polarization [60].

Tumor-derived exosomes are key mediators of macrophage polarization, a process in which the Hippo pathway is actively involved [61]. Under endoplasmic reticulum stress (ERS), the inactivation of the Hsp90/Hippo pathway axis activates the STAT3 and PI3K/AKT/mTOR pathways, thereby inducing the expression of LncRNA HMMR-AS1 and specific microRNAs, including miR-1246, let-7a, and miR-301a-3p [62]. Additionally, the IRE1/PERK axis modulates exosome secretion either by transporting miR-23a-3p and miR-27a-3p or by directly delivering PD-L1 protein, which activates the PI3K/AKT pathway, inhibits PTEN, and upregulates PD-L1, collectively enhancing M2 polarization [63].

In pancreatic ductal adenocarcinoma (PAAD), tumor cells exploit the Hippo-YAP pathway to stimulate macrophage-derived CCL5 secretion. CCL5, in turn, activates the CCR5/AKT/Sp1/CD44 axis, conferring stemness and chemoresistance to PCCs, thereby promoting tumor progression [23]. Targeting this feedback loop has been shown to improve gemcitabine efficacy in PAAD.

Collectively, the Hippo pathway plays multifaceted roles in tumor immune regulation. By downregulating PD-L1 transcription, relieving tumor-mediated T cell suppression, and promoting chemokine release, it activates the tumor immune microenvironment and facilitates cytotoxic T lymphocyte recruitment, thereby enhancing immunotherapeutic efficacy. These findings provide a compelling rationale for developing Hippo pathway-targeted strategies in cancer immunotherapy (Figure 3C).

## 5. Feedback Regulation of the Hippo Pathway by the TME

The interaction between the TME and the Hippo pathway is bidirectional, with various signaling molecules and physical cues within the TME reciprocally modulating Hippo pathway activity.

### 5.1. Regulation of the Hippo Pathway by Tumor Microenvironment Signaling Molecules

The TME is not a static structure but a dynamic system composed of diverse cell types, ECM, and soluble signaling molecules. These signals—including inflammatory cytokines, growth factors, and metabolic byproducts—profoundly influence tumor cell behavior. Inflammatory cytokines within the TME, such as TNFα and IL-6, activate gp130–Src and downstream kinases, thereby promoting YAP/TAZ expression and nuclear translocation. This enhances their interaction with TEAD transcription factors, driving the transcription of genes that support proliferation and immune suppression [64]. Hypoxic conditions in the TME further facilitate the formation of HIF-1α-unphosphorylated YAP complexes, which cooperatively activate target gene expression to promote tumor cell survival, angiogenesis, and invasive phenotypes under low-oxygen conditions [65].

Mechanical cues, including alterations in ECM stiffness and intercellular forces, suppress the core Hippo pathway kinases MST and LATS via Rho GTPase-dependent mechanisms, allowing unphosphorylated YAP/TAZ to translocate to the nucleus and induce pro-tumorigenic gene programs [66]. YAP/TAZ activation is also regulated by the cadherin/α-catenin complex, which sequesters YAP/TAZ at cell–cell junctions and restricts nuclear translocation. Actomyosin tension and the integrity of the F-actin cytoskeleton serve as rate-limiting steps for YAP nuclear localization and transcriptional activity. Disruption of the actin cytoskeleton or inhibition of Rho/ROCK signaling prevents the nuclear entry of YAP/TAZ even in the presence of ECM adhesion, whereas intact F-actin fibers and cell–ECM coupling facilitate nuclear localization and transcriptional activation. Nuclear YAP/TAZ further upregulates focal adhesion-associated genes, including vinculin, zyxin, talin, and integrin αVβ3, reinforcing the linkage between the actin cytoskeleton, the plasma membrane, and the ECM, thereby stabilizing cellular mechanics and enhancing spreading and migratory capacity [67]. Within the immune microenvironment, YAP/TAZ also upregulate chemokines such as CCL2 and CCL5, as well as the immune checkpoint molecule PD-L1, promoting the infiltration and polarization of MDSCs and tumor-associated macrophages (TAMs), thereby enhancing immune evasion. Increased matrix stiffness further reinforces YAP/TAZ activity, establishing a positive feedback loop that augments CAF function and the tumor-promoting characteristics of the microenvironment [42].

### 5.2. Regulation of the Hippo Pathway by Inflammatory Cytokines

Inflammation is a central component of the TME, with inflammatory cytokines playing dual roles in tumor progression. While they can facilitate immune evasion and metastasis, they may also modulate tumor cell growth and differentiation via the Hippo pathway.

Macrophages are a key immune cell population within the TME, exhibiting high functional plasticity. The polarization state of TAMs profoundly influences tumor progression and metastasis. Interactions between macrophages and PCCs are tightly regulated by cytokines, affecting gemcitabine resistance [66]. Notably, PCC-derived amphiregulin (AREG) activates the Hippo-YAP pathway to induce macrophage secretion of CCL5, which in turn engages the CCR5/AKT/Sp1/CD44 axis in PCCs, conferring stemness and chemoresistance [23].

Pro-inflammatory cytokines such as TNFα promote YAP/TAZ dephosphorylation and nuclear translocation, primarily through Rho GTPase activation and cytoskeletal remodeling that inhibit the upstream Hippo pathway kinases LATS1/2. Nuclear YAP/TAZ then interact with TEAD transcription factors to enhance the expression of target genes, including VCAM1 and ICAM1, thereby promoting inflammatory cell adhesion and immune responses. This axis operates in parallel to, rather than redundantly with, the canonical NF-κB pathway [68]. Additionally, cytokines such as IL-6, via their co-receptor gp130, activate JAK/STAT signaling and other downstream kinases, indirectly suppressing MST1/2 and LATS1/2-mediated phosphorylation of YAP/TAZ. This amplifies YAP/TAZ nuclear activity, driving the transcription of pro-inflammatory and pro-proliferative genes and establishing a positive feedback loop between inflammation and Hippo pathway activation [69].

This regulatory network is not restricted to inflammatory diseases but is equally critical within the TME: cytokine-driven upregulation enhances YAP/TAZ-mediated immunoregulatory gene expression, such as CCL2 and CSF1, promoting the infiltration of immunosuppressive cells. Sustained YAP/TAZ activity further stimulates the production of inflammatory cytokines, including IL-6, generating a pro-tumorigenic inflammatory circuit. These mechanisms provide a strong theoretical foundation for therapeutic strategies targeting the inflammation–Hippo pathway axis.

### 5.3. Regulation of the Hippo Pathway by Growth Factors

During cell proliferation and tumorigenesis, multiple growth factors modulate cell fate decisions by regulating the activity of the core Hippo pathway effectors YAP and TAZ. Epidermal growth factor (EGF) and TGF-β have been shown to influence the Hippo pathway through diverse mechanisms [70]. In Epstein–Barr virus (EBV)-associated gastric cancer, viral infection induces the expression and secretion of OLFM4, which binds FAT1 and disrupts its interaction with MST1, thereby activating YAP signaling and promoting tumor progression.

Classical EGF signaling through its receptor EGFR activates the PI3K–PDK1 and RAS–MAPK cascades, suppressing the phosphorylation activity of upstream Hippo pathway kinases LATS1/2. This reduces the phosphorylation of YAP at Ser127, allowing dephosphorylated YAP/TAZ to accumulate in the nucleus, associate with TEAD transcription factors, and drive the expression of pro-proliferative and anti-apoptotic genes such as CTGF and CYR61. By integrating extracellular signals into the Hippo pathway, growth factors like EGF finely regulate cellular proliferation [71].

TGF-β also modulates the Hippo pathway through its canonical SMAD axis, which remodels the extracellular matrix and alters cell morphology. These changes indirectly affect the activity of upstream mechanical sensors and Hippo pathway kinases, thereby influencing YAP/TAZ subcellular localization and transcriptional activity to promote cell migration and EMT. Other growth factors, including IGF and HGF, activate PI3K/AKT signaling via receptor tyrosine kinases (RTKs), suppressing MST1/2 and LATS1/2 activity and enhancing nuclear YAP/TAZ localization and target gene expression, ultimately driving cell proliferation, survival, and motility [72].

This interplay between growth factors and the Hippo pathway underscores its function as a central integrator of extracellular cues, providing a critical mechanism by which tumor cells convert exogenous growth signals into sustained proliferative and invasive phenotypes.

### 5.4. Regulation of the Hippo Pathway by Metabolites

Cellular metabolites, as direct outputs of intracellular energy states and nutrient availability, regulate the core Hippo pathway effectors YAP/TAZ through multiple molecular mechanisms, thereby finely coordinating metabolism with proliferation, differentiation, and other cellular functions. Glucose metabolism exerts a direct influence on the Hippo pathway. Under high-glucose conditions, enhanced glycolysis and oxidative phosphorylation increase the ATP/AMP ratio, suppressing activation of the energy sensor AMPK. This reduces both the direct phosphorylation of YAP/TAZ by AMPK and AMPK-mediated activation of LATS1/2, allowing YAP/TAZ to remain dephosphorylated, translocate to the nucleus, and drive the expression of pro-proliferative genes [73]. Conversely, energy stress or glucose deprivation activates AMPK, which directly or indirectly enhances LATS1/2-mediated phosphorylation of YAP/TAZ, leading to their cytoplasmic retention and inactivation. This mechanism translates cellular energy status into Hippo pathway-mediated cell fate signals.

Lipid metabolites influence the Hippo pathway axis through multiple pathways. For example, isoprenoid intermediates such as geranylgeranyl pyrophosphate (GGPP), produced via the classical mevalonate pathway, serve as essential substrates for Rho GTPases. Activation of Rho promotes cytoskeletal reorganization and inhibits LATS kinase activity, facilitating the nuclear localization and transcriptional activity of YAP/TAZ [74]. Statins, by inhibiting HMG-CoA reductase and reducing mevalonate-derived metabolites, diminish the nuclear accumulation of YAP/TAZ and downstream gene expression. Other metabolic intermediates, including fatty acids and carbohydrate derivatives, modulate Hippo pathway activity indirectly through F-actin remodeling and GPCR signaling. Metabolic cues not only regulate upstream kinases such as AMPK and mTOR to influence LATS1/2-mediated inhibitory phosphorylation of YAP/TAZ but also modulate Rho GTPase and cytoskeletal dynamics to fine-tune Hippo pathway output [75].

CAFs, another key component of the TME, produce substantial lactate via glycolysis. CAF-derived lactate can prevent MST1 ubiquitination and degradation, thereby promoting stemness in oral squamous cell carcinoma [76]. Specifically, lactate exposure upregulates DLG5, which increases the expression of the E3 ubiquitin ligase Cullin 3, enhancing MST1 ubiquitination and degradation and consequently activating YAP signaling [39].

Glutamine, a critical energy and nitrogen source for tumor cells, is tightly linked to the Hippo pathway. Tumor dependence on glutamine, or “glutamine addiction,” modulates tumor phenotypes and the microenvironment via the Hippo/YAP and mTORC1 pathways [77]. Targeting glutamine metabolism—through inhibition of the glutamine transporter ASCT2 or glutaminase GLS1—represents a promising therapeutic strategy.

## 6. Drug Development Targeting the Hippo Pathway

Currently, no Hippo pathway-targeted drugs have been approved for clinical use. Ongoing clinical development focuses on TEAD palmitoylation inhibitors and agents that disrupt YAP-TEAD interactions, primarily for advanced solid tumors, particularly mesotheliomas harboring NF2/LATS mutations or YAP fusions, with emerging strategies exploring combinations with immunotherapy and targeted therapies. The Hippo pathway plays a pivotal role in tumorigenesis, and aberrant activation of its key transcriptional effectors, YAP/TAZ, is closely associated with cancer initiation, progression, and therapy resistance. Consequently, the development of YAP/TAZ inhibitors, as well as combination strategies with conventional therapies such as chemotherapy and immunotherapy, holds significant clinical potential.

### 6.1. TEAD Palmitoylation Inhibitors

Mutations or functional aberrations in key Hippo pathway regulators, such as NF2 and LATS1/2, are central drivers of sustained activation of the YAP/TAZ-TEAD axis. Direct targeting of YAP/TAZ is challenging due to their intrinsically disordered structure and lack of small-molecule binding pockets, while conventional YAP-TEAD interface inhibitors suffer from low affinity and poor selectivity owing to broad, shallow, and highly hydrophilic interaction surfaces. In contrast, the highly conserved auto-palmitoylation of TEAD represents a critical breakthrough in overcoming these druggability challenges and provides a solid theoretical foundation for the development of TEAD palmitoylation inhibitors [78].

TEAD auto-palmitoylation is essential for its transcriptional activity. In human TEAD1-4, conserved cysteine residues within the core domain enable self-palmitoylation without requiring an acyltransferase. The palmitoyl chain covalently binds via a thioester linkage and embeds into a specific hydrophobic pocket, promoting protein folding and stability while remodeling the YAP/TAZ binding interface. This ensures efficient assembly and transcriptional activity of the YAP/TAZ-TEAD complex, without disrupting TEAD binding to the endogenous tumor suppressor VGLL4, thereby providing a therapeutic window for selective inhibition [79].

Most TEAD palmitoylation inhibitors are small-molecule hydrophobic compounds that competitively occupy the TEAD palmitoylation pocket, preventing palmitoyl-CoA binding and auto-palmitoylation. This triggers a cascade of anti-tumor effects: disrupting TEAD conformational stability, dramatically reducing its affinity for YAP/TAZ, and blocking the formation of oncogenic transcriptional complexes [80]. Unpalmitoylated TEAD undergoes conformational destabilization and proteasomal degradation, decreasing nuclear TEAD levels. The net result is comprehensive suppression of YAP/TAZ-TEAD-mediated oncogenic transcription, inhibition of tumor cell proliferation, induction of apoptosis, blockade of invasion and metastasis, and reversal of fibroblast-mediated immunotherapy resistance within the tumor microenvironment, thereby enhancing the efficacy of immune checkpoint inhibitors.

TEAD palmitoylation inhibitors offer superior druggability, high target specificity, and precise therapeutic efficacy, directly targeting the terminal oncogenic node of the pathway and bypassing the complexity of upstream regulation. Patient stratification based on biomarkers such as NF2/LATS mutations, nuclear YAP localization, or YAP fusions may further optimize clinical benefit. These inhibitors have progressed from preclinical studies into phase I/II clinical trials, with some candidates demonstrating clear anti-tumor activity in malignant mesothelioma. As a result, TEAD palmitoylation inhibition represents the most promising translational strategy in Hippo pathway-targeted oncology. Future optimization through isoform selectivity and combination therapy strategies may reduce off-target toxicity, overcome monotherapy resistance, and provide precision therapeutics for tumors with aberrant Hippo pathway activation.

### 6.2. YAP/TEAD Interaction Inhibitors

The YAP-TEAD interaction is an essential downstream node for oncogenic signal output in the Hippo pathway, with the interface encompassing multiple conserved functional regions that underpin complex stability and transcriptional activity. Compared to the inherent druggability challenges of directly targeting YAP/TAZ, the YAP-TEAD interface, despite its broad, shallow, and hydrophilic nature, can be selectively inhibited, offering promising therapeutic potential [60].

In contrast to TEAD palmitoylation inhibitors, which act through post-translational modification, YAP-TEAD disruptors directly target the protein–protein interaction. Some compounds precisely engage the core YAP-TEAD interface, mimicking key YAP binding motifs to occupy the TEAD binding site and prevent the physical association of YAP/TAZ with TEAD, thereby blocking complex assembly at its origin. For example, Super-TDU directly mimics the TEAD-binding domain of YAP, competitively displacing bound YAP with higher affinity and rapidly suppressing the transcription of downstream target genes [81]. Other agents act allosterically, binding to distal sites on TEAD, such as the lipid pocket, to induce irreversible conformational changes that distort the YAP-binding surface, reduce binding affinity, and prevent the formation of a stable, functional complex. These allosteric inhibitors combine high selectivity with favorable druggability and represent the mainstream direction in current small-molecule development [82].

Although no YAP-TEAD interaction inhibitors have yet been approved globally, multiple candidates from both mechanistic classes have entered clinical trials (Table 1). Future development will focus on isoform-selective optimization, minimization of off-target toxicity, exploration of combination therapy strategies, and biomarker-guided patient stratification, with the goal of establishing these agents as standardized targeted therapies for tumors with aberrant Hippo pathway activation.

## 7. Conclusions and Perspectives

The Hippo pathway serves as a central hub coordinating cell fate decisions and interactions with the TME. Its canonical regulation relies on the MST1/2-SAV1-MOB1A/B-LATS1/2 kinase cascade to control the phosphorylation status of YAP/TAZ, while the nuclear translocation of YAP/TAZ and their association with TEAD transcription factors constitute the critical node mediating oncogenic signal output [83]. The pathway does not act in isolation but integrates with multiple signaling cascades, including EGFR, Wnt/β-catenin, and Notch, and incorporates microenvironmental cues such as mechanical stress, cell density, and metabolic status, forming a multilayered, networked regulatory system that plays central roles in tumor initiation and progression [8]. Aberrant activation of YAP/TAZ directly drives tumor cell proliferation, inhibits apoptosis, maintains cancer stem cell traits, and orchestrates metabolic reprogramming via glutamine and lipid metabolism, providing the energetic and biosynthetic foundation for rapid tumor growth [84].

Dysregulation of the Hippo pathway mediates chemoresistance and targeted therapy resistance by modulating tumor–microenvironment interactions. Examples include enhanced gemcitabine resistance in pancreatic cancer through tumor cell–macrophage positive feedback loops and drug resistance in intrahepatic cholangiocarcinoma caused by SAV1 mutations [85]. The Hippo pathway also regulates CAF activation, heterogeneity, and extracellular matrix remodeling, shaping a tumor-promoting stromal microenvironment. Concurrently, it establishes an immunosuppressive TME by upregulating PD-L1, inhibiting CD8^+^ T cell function, promoting M2 macrophage polarization, and modulating chemokine secretion, thereby facilitating immune evasion. Conversely, microenvironmental cues—such as inflammatory cytokines, growth factors, metabolites, and physical signals—can feedback to modulate Hippo pathway activity, forming a “microenvironmental signal–Hippo pathway-malignant phenotype” positive feedback loop that further promotes tumor progression [86].

Direct pharmacological targeting of YAP/TAZ remains challenging. Current strategies focus on TEAD palmitoylation inhibitors and YAP/TEAD interaction disruptors, with several candidates advancing into clinical trials. Despite significant progress in understanding the Hippo pathway in cancer and in drug development, the pathway’s regulatory complexity, TME heterogeneity, and clinical translation hurdles remain major challenges. Future research integrating single-cell sequencing, spatial transcriptomics, and related technologies to map Hippo pathway activity across cellular subpopulations within tumors will provide new insights for identifying therapeutic targets. Optimizing Hippo pathway-targeted drug design, exploring combination and personalized therapy strategies, and developing precise biomarkers and companion diagnostics may offer novel clinical avenues to improve outcomes for cancer patients.

## Figures and Tables

**Table 1:** Summary of Hippo pathway-targeted therapeutics in clinical development.

Drug	Mechanism	Developer	Clinical Phase	Indication	Safety Profile	NCT Number
VT3989	Potent inhibitor of TEAD protein palmitoylation	Vivace Therapeutics	Phase I/II	Malignant pleural mesothelioma, NF2-mutant solid tumors including non-small cell lung cancer	Well tolerated; primarily grade 1–2 adverse events (proteinuria, peripheral edema, fatigue); proteinuria reversible with dose adjustment	NCT04665206
IK-930	Selective TEAD1 palmitoylation inhibitor	Ikena Oncology	Phase I	Epithelioid hemangioendothelioma, NF2-deficient mesothelioma, meningioma	Well tolerated; lower proteinuria incidence than pan-TEAD inhibitors, supporting a wider therapeutic window	NCT05228015
IAG933	YAP-TEAD interaction inhibitor	Novartis	Phase I (terminated)	Malignant pleural mesothelioma, NF2/LATS1/2 mutant tumors, YAP/TAZ fusion-positive tumors	Moderate tolerability; QT prolongation and proteinuria reported	NCT04857372
ION537	YAP1 mRNA degrader	Ionis Pharmaceuticals	Phase I	HCC, head and neck cancer, NF2-mutant mesothelioma	Dose escalation stage	NCT04659096
ISM6331	Reversible TEAD1/2/3/4 palmitoylation pocket binder	Insilico Medicine	Phase I	Metastatic malignant pleural mesothelioma, NF2-deficient solid tumors	Dose escalation stage	NCT06566079
SIGX2649	Dual mechanism: inhibits palmitoylation and promotes VGLL4 binding	Signet Therapeutics	Preclinical (IND preparation)	Malignant pleural mesothelioma (NF2 loss/LATS mutation), YAP-activated HCC	IND submission in progress	Preparation for IND submission
IAG933	YAP/TEAD allosteric inhibitor	Novartis	Phase I	Advanced malignant pleural mesothelioma (NF2 loss/mutation)	Clinical data not disclosed	NCT04857372
Verteporfin	Direct YAP–TEAD protein interaction inhibitor	Original: QLT Inc.; Global/Commercial: Novartis	Ophthalmology approved; Phase I/II oncology	YAP/TAZ-activated solid tumors (GBM, pancreatic cancer, breast cancer skin metastases, mesothelioma)	Phase I/II trials evaluating safety and preliminary efficacy; no phase III data	NCT04590664 (recurrent GBM, liposomal), NCT03033225 (unresectable pancreatic cancer, PDT combination), NCT02939274 (breast cancer skin metastases, low-dose PDT)
BPI-460372	Highly selective TEAD1/3/4 palmitoylation inhibitor	Betta Pharmaceuticals	Phase I	Malignant mesothelioma, epithelioid hemangioendothelioma	Well tolerated	NCT05789602
SW-682	Pan-TEAD palmitoylation inhibitor	SpringWorks Therapeutics	Phase I (terminated)	Malignant mesothelioma including NF2-mutant tumors	Safety data not disclosed	NCT06251310
TY-1054	TEAD palmitoylation pocket inhibitor	TYK Medicines	Phase I/II	Malignant mesothelioma (NF2 mutant)	Data not yet disclosed	NCT06251310

## Data Availability

No new data were created or analyzed in this study.

## Abbreviations

The following abbreviations are used in this manuscript:

TME	tumor microenvironment
CAFs	cancer-associated fibroblasts
PD-L1	programmed death-ligand 1
MST1/2	mammalian STE20-like kinase 1/2
SAV1	scaffold protein Salvador homolog 1
LATS1/2	large tumor suppressor 1/2
YAP/TAZ	Yes-associated protein/transcriptional co-activator with PDZ-binding motif
TEAD1-4	domain family member 1-4
CSC	cancer stem cell
pEMT	epithelial–mesenchymal transition
TGF-β	transforming growth factor-β
PCCs	pancreatic cancer cells
HCC	hepatocellular carcinoma
ECM	extracellular matrix
myCAF	myofibroblastic CAF
iCAF	inflammatory CAF
apCAF	antigen-presenting CAF
rCAF	regulatory CAF
DCs	dendritic cells
CTLs	CD8^+^ cytotoxic T lymphocytes
MDSC	myeloid-derived suppressor cell
ERS	endoplasmic reticulum stress
PAAD	pancreatic ductal adenocarcinoma
TAMs	tumor-associated macrophages
AREG	PCC-derived amphiregulin
EGF	epidermal growth factor
EBV	Epstein–Barr virus
RTKs	receptor tyrosine kinases
GGPP	geranylgeranyl pyrophosphate
